# Supplementary material for: REIV-TOXO Project: Results from a Spanish cohort of congenital toxoplasmosis (2015–2022). The beneficial effects of prenatal treatment on clinical outcomes of infected newborns
Source: PLoS Negl Trop Dis. 2024 Oct 22;18(10):e0012619. doi: 10.1371/journal.pntd.0012619 (PMC11530059; doi:10.1371/journal.pntd.0012619)
Supplement: S1 Appendix — (DOCX) [file pntd.0012619.s001.docx]

Supporting information

**S1 Appendix. Association between prenatal treatment and newborn characteristics including the “uncertain prenatal treatment group” (7-27 days of treatment) in the analysis within the prenatal treatment group.**

|  | **Prenatal**  **treatment** | | | | **Univariate**  **analysis** | | **Multivariate analysis** | |
| --- | --- | --- | --- | --- | --- | --- | --- | --- |
|  | No (n=19) | | Yes (n=37) | |  |  |  |  |
|  | **n** | **%** | **n** | **%** | **p** | **OR (95% CI)** | **p** | **OR (95% CI)** |
| Confirmed maternal infection |  |  |  |  |  |  |  |  |
| 1^st^ trimester | 3/15 | 15.78 | 2/36 | 5.55 |  |  |  |  |
| 2^nd^ trimester | 1/15 | 5.26 | 10/36 | 27.02 |  |  |  |  |
| 3^rd^ trimester | 11/15 | 57.89 | 24/36 | 64.86 | 0.16 |  |  |  |
| Prematurity | 5/19 | 26.32 | 3/37 | 8.11 | 0.08 |  |  |  |
| Symptomatic CT (all) | 11/19 | 57.89 | 10/37 | 27.03 | **0.03** | **3.71 (1.12–12.21)** | 0.09 | 3.11 (0.80–12.08) |
| Ocular CT | 5/19 | 26.32 | 4/37 | 10.81 | 0.15 |  | 0.19 |  |
| CNS CT | 9/19 | 47.37 | 6/37 | 16.22 | **0.01** | **4.65 (1.28–1678)** | 0.17 |  |
| Generalized CT | 3/19 | 15.78 | 1/37 | 2.70 | 0.11 |  | 0.21 |  |
| New IRC (all) | 6/19 | 31.58 | 2/37 | 5.40 | **0.02** | **8.07 (1.38–47.02)** | **0.01** | **18.55 (1.78**–**192.43)** |
| New ocular IRC | 4/19 | 21.05 | 1/37 | 2.70 | 0.05 | 9.60 (0.93**–**98.13) | **0.04** | **16.85 (1.14–248.16)** |
| New CNS IRC | 3/19 | 15.79 | 1/37 | 2.70 | 0.11 |  | 0.08 | 11.92 (0.70–200.46) |

*CI = confidence interval; CNS = central nervous system; CT = congenital toxoplasmosis; IRC = infection-related complications; OR = odds ratio.*
