# Supplementary material for: REIV-TOXO Project: Results from a Spanish cohort of congenital toxoplasmosis (2015–2022). The beneficial effects of prenatal treatment on clinical outcomes of infected newborns
Source: PLoS Negl Trop Dis. 2024 Oct 22;18(10):e0012619. doi: 10.1371/journal.pntd.0012619 (PMC11530059; doi:10.1371/journal.pntd.0012619)
Supplement: S1 Acknowledgments — (DOCX) [file pntd.0012619.s002.docx]

Supporting information

**S2 Appendix. Members of the Spanish REIV-TOXO group**

Miguel Sánchez (Hospital Universitario Torrecárdenas); Leticia Martínez (Hospital Universitario Torrecárdenas); Rakel Angulo (Hospital de Poniente); Almudena Alonso (Hospital Universitario Puerta del Mar); Victoria Ramos (Hospital de Jerez de la Frontera); Beatriz Ruiz (Hospital Universitario Reina Sofía de Córdoba); Marta Cruz (Hospital de Montilla); José María Gómez (Hospital Universitario San Cecilio); Juan Luís Santos (Hospital Universitario Virgen de las Nieves); Francisco de Borja (Hospital Juan Ramón Jiménez); Juan Salvador Vílchez (Complejo Hospitalario de Jaén); David Moreno and Begoña Carazo (Hospital Regional Universitario de Málaga); Antonio José Conejo (Hospital Vithas Xanit Internacional); Margarita Rojas (Hospital Quirónsalud Marbella); David López (Hospital Costa del Sol); María José Muñoz (Hospital Universitario Virgen de la Macarena); Lola Falcón (Hospital Universitario Virgen del Rocío); Ángela Hurtado (Hospital Sagrado Corazón de Sevilla); Pedro de Paúl (Hospital de Ceuta); María Sánchez (Hospital de Melilla); Matilde Bustillo and Pilar Abenia (Hospital Universitario Miguel Servet); Pilar Collado (Hospital Clínico Universitario Lozano Blesa); Teresa Crespo (Hospital Universitario de Cabueñes); Sonia Lareu (Hospital Universitario Central de Asturias); Pascual Escrivá (Hospital Virgen de los Lirios); Caridad Tapia (Hospital General Universitario de Alicante); María Jesús Ferrández (Hospital General Universitario de Elche); Manuel Roig (Hospital de la Vega Baja); César Gavilán (Hospital Universitario de Sant Joan); Marta Dapena and Vicente Posadas (Hospital General Universitario de Castellón); Ángel González (Hospital Universitario la Ribera de Alzira); José Vicente Arcos and Inma Vilaplana (Hospital Francesc de Borja de Gandia); Blanca Garrido (Hospital de Manises); Elena Montesinos (Hospital General Universitario de Valencia); Ana Pineda (Hospital Universitari Doctor Peset); Rafael Bretón (Hospital Clínic Universitari de Valencia); Manuel Oltra and David Bernad (Hospital General Universitari i Politècnic La Fe); José Cambra (Hospital Lluis Alcanyis); Dara Boza (Hospital Dr. José Molina Orosa); Elena Colino (Complejo Hospitalario Universitario Insular Materno Infantil); Sara Díaz (Complejo Hospitalario Universitario de Canarias); Dolores Sabina (Hospital Universitario Nuestra Señora de la Candelaria); Beatriz García (Hospital Universitario Marqués de Valdecilla); Elena Gil (Hospital Comarcal de Laredo); Juan Arnáez and María Cristina de Frutos (Complejo Asistencial Universitario de Burgos); Sandra Terroba (Complejo Asistencial Universitario de León); María Paz Barrio (Complejo Asistencial Universitario de Palencia); Rubén García (Complejo Asistencial Universitario de Salamanca); Miriam Hortelano (Complejo Asistencial de Segovia); Asunción Pino (Hospital Clínico Universitario de Valladolid); Raquel Izquierdo (Hospital Universitario del Río Hortega); Marta Pareja and María del Carmen Manzanero (Complejo Hospitalario Universitario de Albacete); Ana Muñoz Serrano and Elena Resa (Hospital General la Mancha Centro de Alcazar de San Juan); María José Hernández and Ana Aldea (Hospital General Universitario de Guadalajara); Yolanda López (Complejo Hospitalario Universitario de Toledo); María Mendez (Hospital Universitari Germans Trias i Pujol); Mª Ángeles López-Vílchez

(Hospital del Mar); Romina Conti (Corporació Sanitària Parc Taulí); Elisenda Moliner (Hospital de la Santa Creu i Sant Pau); Antoni Noguera (Hospital Sant Joan de Déu); Berta Pujol (Hospital General de Granollers); Zulema Lobato (Xarxa Assistencial Sanitària de Manresa); Roser Díez (Hospital de Mataró); Laura Castells (Hospital Universitari General de Catalunya); Marina Fenoy (Consorci Sanitari de Terrassa); Alicia Mirada (Hospital Universitari Mútua de Terrassa); Grisel Vilagrasa (Hospital Universitari Dexeus); Isabel Vives (Hospital Quirónsalud Barcelona); Montse Ruiz (Hospital Universitari de Vic); María del Mar Peñas (Hospital Sant Jaume de Calella); Francesc Ripoll (Hospital Santa Caterina); Laura Geronès (Hospital de Palamós); Marcelina Algar (Hospital de Figueres); Antoni Foguet (Hospital Sant Jaume d’Olot); María Rocío Vilchez (Hospital de Campdevànol); Xavier Bringué (Hospital Universitari Arnau de Vilanova); Neus Rius (Hospital Universitari Sant Joan de Reus); Olga Calavia (Hospital Universitari de Tarragona Joan XXIII); Silvia Franch (Hospital de Tortosa Verge de la Cinta); Elena del Castillo (Complejo Hospitalario Universitario de Badajoz); Mercedes García (Complejo Hospitalario de Mérida); María Casero (Complejo Hospitalario Universitario de Cáceres); Rosa Romaris (Complejo Hospitalario Universitario de Ferrol); Irene Rivero (Hospital Clínico Universitario de Santiago); Santiago García (Complejo Hospitalario Universitario de Ourense); Jose Couceiro (Complejo Hospitalario Universitario de Pontevedra); Luisa Gonzalez (Hospital Álvaro Cunqueiro de Vigo); Susana Herrero (Hospital Universitario Son Llàtzer); Ana López (Hospital Universitario Son Espases); Amelia Sánchez (Hospital Can Misses); Inés Esteban (Hospital de San Pedro); Elvira Cobo (Hospital Universitario Fundación de Alcorcón); Pilar Galán (Hospital Universitario de Fuenlabrada); Irene Cuadrado (Hospital de Getafe); María Luz García (Hospital Universitario Severo Ochoa); Alicia Hernanz (Hospital General Universitario Gregorio Marañón); José Tomás Ramos (Hospital Universitario Clínico San Carlos); Paula Rodríguez (Hospital Universitario La Paz); María José Cilleruelo (Hospital Universitario Puerta de Hierro); Ana Vidal (Hospital Universitario de Móstoles); Gema Sabrido (Hospital Universitario Rey Juan Carlos); Julia Jensen (Hospital Universitario Infanta Cristina de Parla); Hemir David and Escobar Pirela (Hospital de Torrejón); Alfredo Tagarro and Teresa Reinoso (Hospital Universitario Infanta Sofía); Loreto García-Trevijano (Hospital de Villalba); Gloria Caro (Hospital Universitario Infanta Elena); José Ramón Fernández (Complejo Hospital Universitario Santa María del Rosell–Santa Lucía de Cartagena); Blanca Rodríguez (Hospital Universitario Rafael Méndez de Lorca); Santiago Alfayate and Miguel Alcaraz (Hospital Clínico Universitario Virgen de la Arrixaca); Asier Oliver (Clínica Universidad de Navarra); Merche Herranz and Andrea Ilundain (Complejo Hospitalario de Navarra); Jorge García (Hospital Universitario Araba); María Itziar Pochevilla (Hospital Universitario de Cruces); Joseba Rementería (Hospital Universitario de Basurto); Eider Oñate (Hospital Universitario de Donostia).
