# Supplementary material for: REIV-TOXO Project: Results from a Spanish cohort of congenital toxoplasmosis (2015–2022). The beneficial effects of prenatal treatment on clinical outcomes of infected newborns
Source: PLoS Negl Trop Dis. 2024 Oct 22;18(10):e0012619. doi: 10.1371/journal.pntd.0012619 (PMC11530059; doi:10.1371/journal.pntd.0012619)
Supplement: Attachment — Submitted filename: Guarch-Ibanez et al. paper_PNTD 2024.docx [file pntd.0012619.s003.docx]

The paper titled "Results from the Spanish cohort of congenital toxoplasmosis (2015-2022)" by Guarch-Ibanez et al. highlights the importance of maternal screening for T. gondii in Spain and treatment for the management of congenital toxoplasmosis. The conclusions drawn from this paper support the implementation of a mandatory prenatal screening program followed by treatment for women who test positive to reduce the impact of the disease on newborns and during follow-up. Here, we applaud the authors for citing on many occasions France, a country with historically successful management of congenital toxoplasmosis, and a similar incidence rate of CT in Spain, which suggests that prenatal screening is cost-effective compared to neonatal screening. This approach also empowers pregnant women to educate themselves on how to reduce the risk of infection and decrease the incidence of toxoplasmosis in the country. However, the data from the Spanish cohort lacks the monthly serological screening of pregnant women, which creates uncertainty about the timing of maternal infection before diagnosis and the potential delay in treatment following seroconversion. Despite this, the paper is well-written and interesting to read. I recommend that the authors emphasize these key points to enhance the presentation of this manuscript.

1. The current title of the manuscript does not accurately represent its content and should be changed.

2. The introduction and conclusion of the manuscript need further development. Please provide more details on why certain European countries (such as the UK, Denmark, and Sweden) and specific regions in Spain have rejected prenatal screening. Some argue that this decision is due to the clinical harms associated with false-positive diagnoses, the low and uncertain benefits of treatment, the harms associated with treatment, and the high costs of prenatal screening.

3. Are there any studies from the Spanish cohort on the genetics of the parasite that could explain the severity observed in certain newborns, such as the case of a newborn with hydrocephalus?

4. Technical information is necessary to understand how the diagnosis and screenings of pregnant women were reported in this cohort. Was the avidity test used to confirm those who are seropositive and not required for the screening? Which confirmation test for IgG/IgM was used? Do all these hospitals use the same confirmatory test? Is there any consensus on the choice of this test?

5. Please provide information on whether pregnant women who tested positive for toxoplasmosis had any adverse pregnancy outcomes, such as miscarriages or preterm births. Did you observe any differences in outcomes between toxoplasmosis-positive and negative women or between those who received treatment and those who did not?

6. The authors should provide evidence to support their claim about the impact of SARS-CoV-2 on the decrease in reported cases of T. gondii infection in mothers in Spain since 2018 and whether similar trends are seen in other congenital diseases. Please include any relevant references from the literature.

7. It would be helpful to enhance the discussion by emphasizing the importance of new diagnostic tests (e.g. rapid tests) that can be incorporated into prenatal screening to alleviate the challenges of monthly screening in a prospective study of this cohort.
