# Supplementary material for: REIV-TOXO Project: Results from a Spanish cohort of congenital toxoplasmosis (2015–2022). The beneficial effects of prenatal treatment on clinical outcomes of infected newborns
Source: PLoS Negl Trop Dis. 2024 Oct 22;18(10):e0012619. doi: 10.1371/journal.pntd.0012619 (PMC11530059; doi:10.1371/journal.pntd.0012619)
Supplement: Attachment — Submitted filename: Guarch-Ibanez et al. paper_PNTD 2024 _response to reviewers.docx [file pntd.0012619.s004.docx]

Reviewer's Responses to Questions

In this document we gather our responses to all reviewer´s comments, they are made in blue after every comment or question.

<b>Key Review Criteria Required for Acceptance?</b></br></br>
As you describe the new analyses required for acceptance, please consider the following:</br></br>

<b>Methods</b></br></br>
-Are the objectives of the study clearly articulated with a clear testable hypothesis stated?</br>
-Is the study design appropriate to address the stated objectives?</br>
-Is the population clearly described and appropriate for the hypothesis being tested?</br>
-Is the sample size sufficient to ensure adequate power to address the hypothesis being tested?</br>
-Were correct statistical analysis used to support conclusions?</br>
-Are there concerns about ethical or regulatory requirements being met?</br></br>

Reviewer #1: −  The exhaustivity of this cohort must be doubted given that 22% of diagnosed cases were not included. At least, regional data about the parasite strain types in human disease and the prevalence of seropositivity should be provided along with the number of life births per year during the same period (also to be added to figure 1).

Toxoplasma genotyping is a complex procedure which requires obtaining human isolates and can only be performed in highly specialised laboratories in Spain. In our study, genotyping was not feasible due to the lack of an established sample processing protocol.

The study of seropositivity in pregnant women is not part of the aim of our study. There are no recent data on seroprevalence in Spain after 2015.

−       That 16 children with confirmed CT could not be included in the registry limits representativity. Attempts should be made to get records of these children, at least a root data set to demonstrate that these were similar in the severity of affection to the included patients.

We acknowledge that the exclusion of the 16 children with confirmed CT from the registry limits representativity. However, the characteristics of REIV-TOXO, a voluntary registry, where the investigators at each site are responsible for entering the data, limit the authors' access to all cases, and thus there are 16 cases that were never entered despite our efforts to contact the collaborators on repeated occasions. Additionally, without parental consent, we are unable to provide this information. Despite this, our registry represents the highest number of cases registered in Spain, much higher than those reported by the public health authorities.

−       What seems worrisome to me is the duration of treatment during pregnancy. Once the decision in favour of treatment is made, any established guideline clearly states the need for maternal treatment until birth, while in this series, mothers were categorized as having received treatment if they had received it for at least 7 days (line 157). Ther is neither clinical nor experimental evidence that a treatment of such a short duration is of any impact to the fetus, and namely not for preventing infection. I have no clue, how the authors want to deal with this point. Personally, I would choose a minimal treatment duration of 4 weeks for tailoring mothers to any group, or provide 3 groups (for the price of a further reduction of statistical power) with one group with treatment up to 7 days (no treatment group), one with longer intercurrent treatment, and finally one with treatment from diagnosis until delivery, which represents the recommended standard, once a decision to treat has been made. Generally, a median time of 37 days under treatment is away from treatment standards and a very critical point. This might well explain why the portion of children with cerebral affections was remarkably high compared to other cohorts (line348-52). Moreover, possibly, the number of children with ocular manifestation of their CT is relatively low so that the experience of the ophthalmologists in examining newborns with CT was granted. The merely unpigmented lesions at birth are definitely easily missed, even in experienced hands.

We agree with the reviewer that there is uncertainty regarding the potential relationship between the duration of prenatal treatment and its possible effects on the fetus. Therefore, we have redone the statistical analysis and have identified three categories (lines 140-144):

-Prenatal treatment: defined as receiving one or both treatments during pregnancy for at least 28 days.

-Uncertain prenatal treatment: treatment lasting between 7 and 27 days.

-No treatment: treatment less than 7 days

Because no treatment is not the same as a few days of treatment, with an unknown impact on the course of fetal infection, we decided to exclude the uncertain prenatal treatment group from the statistical analysis. It is important to note that early discontinuation of prenatal treatment was due to the end of pregnancy in all 8 cases within this group (lines 217-218). Interestingly, the beneficial effects of prenatal treatment remained unchanged when prenatal treatment lasting more than 7 days was included in the analysis in the treatment group, as mentioned in the Discussion (lines 331-334).

In relation to the proportion of cases with neurological and ocular involvement, the decrease in the number of ocular forms detected at birth is likely due to the lower number of cases diagnosed because of the lack of prenatal screening and the characteristics of REIV-TOXO. As we mention in the Discussion, REIV-TOXO does not provide a full picture of CT in Spain and may not have detected all cases, but it does show a significant sample of toxoplasmosis in Spain, and as you mention, in Europe. When comparing our data with those published in the French cohort (2015-2021), no differences in the percentages of moderate and severe cases are observed between the two cohorts:

|  | French cohort n, (%) | REIV-TOXO n, (%) | p |
| --- | --- | --- | --- |
| Moderate forms | 65 (75%) | 14 (70%) | 0.66 |
| Severe forms | 22 (25%) | 6 (30%) | 0.66 |

Moderate forms include: peripheral retinochoroiditis, intracranial calcifications and generalized disease.

Severe forms include: macular retinochoroiditis, seizures, hydrocephalus, microcephaly, brain parenchymal lesions, intraventricular hemorrhage, and involvement of 2 or more features of the moderate form.

−       A delay of 11 days between diagnosis and treatment is short, but not meaningful, if the time of contamination cannot be estimated. Therefore, more detailed information about the gestational situation at contamination might be provided if possible.

We agree with the reviewer that if serological screening is performed once each trimester, there is greater uncertainty about the time of maternal infection. Unfortunately, we cannot provide any more information on the timing of maternal infection than is already given in the manuscript. In the case of monthly screening strategy, the margin of error at the time of maternal diagnosis is smaller, but it is also possible that it may exceed the “window of opportunity” period. We mention this limitation in the Discussion section (lines 347-354). In our opinion, this limitation exists to a greater or lesser extent in all studies evaluating the effect of prenatal treatment of CT, but it does not invalidate their results in favor of prenatal treatment.

−       Since the topic of this paper is to assess the impact of prenatal screening and treatment, the case diagnosed during the 1st year of life should be excluded from the analysis not having received either of both (line 220).

In our opinion, these cases should not be excluded. As explained in the Methods section, the analysis is based on whether prenatal treatment was received or not, rather than on whether screening was performed. Our goal is to demonstrate the importance of treatment and, by extension, the significance of screening. If these cases, which were symptomatic and severe, are excluded, the apparent effect of treatment and screening would be minimized, a mistake made by previous studies.

−       Random screening and systematic screening data must not be pooled. I would recommend to differentiate pregnancies where a systematic screening was performed (at least 1x per trimester) and those without a systematic screening to make sure the interval between infection and its detection can be estimated; please add systematic screening to reporting as an independent confounder with systematic screening possibly being more relevant than unsystematic reporting (line 94).

As we show in the manuscript, the trimester of maternal infection was established in the majority of pregnancies (89/54 mothers, 90.7%) because screening was done every trimester. Unfortunately, we are not able to differentiate between trimester-based screening (90.7%) and random screening (9.3%) where the exact timing of maternal seroconversion was not estimated.

−       The application of a logistic regression model is not meaningful as long as the time gap between infection and treatment initiation and the duration of treatment are not included. Any results regarding regimen and outcomes are therefore useless and need to be removed.

It is another limitation of our study that we have introduced in the Discussion (lines 404-406). We could not assess the multivariate analysis to investigate any possible association between prenatal treatment and outcomes adjusted by the time gap between maternal infection and prenatal treatment (“window of opportunity”) due to a very small sample size. Despite all limitations, our results suggest that children who received prenatal treatment had better outcomes at birth and during follow-up.

Reviewer #2: I have doubts about the definition of the population of infected newborns

The definition of infected newborns follows the criteria
The sample size is sufficient
No ethical concerns
There are no concerns regarding compliance with ethical or regulatory requirements

We conducted a retrospective and prospective observational multicentre study of children with confirmed CT recorded in the national REIV-TOXO database between 1 January 2015 and 30 June 2022. Our criteria of confirmed CT are in line with the established criteria in the literature:

**Confirmed congenital *Toxoplasma* infection** – Congenital infection is confirmed by **any** of the following:

1-Positive IgG with positive IgM and/or IgA – A positive *Toxoplasma* IgM and/or IgA

2-Positive polymerase chain reaction (PCR) in blood, CSF, urine, amniotic fluid or placenta.

3-Increase in anti-*Toxoplasma* IgG titer during the first year of life

4-Positive IgG beyond 12 months of age

Reviewer #3: the study design is appropriate to address the stated objectives
the population clearly described and appropriate for the hypothesis being tested
the sample size is sufficient to ensure adequate power to address the hypothesis being tested

statistical analysis is correct to make any conclusion

--------------------

<b>Results</b></br></br>
-Does the analysis presented match the analysis plan?</br>
-Are the results clearly and completely presented?</br>
-Are the figures (Tables, Images) of sufficient quality for clarity?</br></br>

Reviewer #1: −  Given that only 54 pregnancies were registered from 122 hospitals over a period of 7 years implies that either a significant number of pregnancy-associated cases of toxoplasmosis have been missed or that the problem is on the decline: a threefold reduction in the number of cases per year is not in favour of a significant health concern due to CT in Spain. On the other hand, each single case with organ damage that can be prevented is a life changer for the affected families. As the authors mention, a systematic screening was not performed, which means that their cohort may be a selection of more severely affected pregnancies or at least an uncertainty of the obstetricians triggering maternal blood testing. This is indicated by a higher than expected prevalence of symptomatic children (table 3).
−       As long as not proven different, the data may be representative of a European cohort, which needs to be stated in line 85.

In line 85 we mention the importance of the clinical manifestations of CT, but not the representativity of our cohort from an epidemiological perspective. Accordingly, we have added your suggestion in line 298.

−       Generally, the parasites have a tropism to the neuro-epithelium, which means they primarily affect the retina and only secondarily spread to the choroid. Affected patients thus pathophysiologically present a **retinochoroiditis**, but not a chorioretinitis. Please change the wording throughout.

Thank you for the comment. We have made the changes you suggested.

−       Cost coverage may be a relevant factor differentiating systematic from non-systematic screening. Since social situation is an important influence factor, as mentioned by the authors, this point deserves acknowledgement. Mothers receiving full cost coverage should be compared to those paying out of the pocket regarding time between presumed infection and its diagnosis and treatment.

REIV-TOXO does not provide data on the economic costs of CT in infected pregnant women and children, nor is it the purpose of our study to evaluate it from this point of view. It should be noted that the Spanish healthcare system, through the National Health System (NHS), provides free healthcare to everyone.

−       Was the 3-fold reduction in cases recorded between 2019-22 linked to changes in the screening strategy or cost coverage (lines 174-8)? As mentioned, the number of life births needs to be compared during the 2 periods to allow an interpretation of these findings.

As we mentioned in the Discussion (lines 305-313), the progressive withdrawal of prenatal screening for *T. gondii* in some regions of Spain is the main explanation of the reduction of the number of cases. In response to your feedback, we have included the decrease in birth rates as an additional factor contributing to the reduction in the number of cases. Most likely, the reduction in the number of live births between the 2 periods (420,290 live births in 2015 to 337,380 in 2021) may have influenced the reduction in the number of CT cases but to a lesser extent than the other factors outlined in the manuscript.

−       That “Newborns born from mothers treated prenatally had a reduced risk of CT clinical features at birth and a lower risk of further complications during follow-up” is supported by the data, while I do not see a four-fold and an eight-fold lower risk after talking diagnostic and therapeutic differences into account. A portion of 32% of IRC over 24 months in untreated patients, again, is significantly higher than expected from other European series. One reason for this might be that the portion of immigrants from geographical areas with a higher virulence is higher than in not spanish-speaking countries (see also lines 91-2). Are there data around to address this impact factor? The sentence (lines 286-7) is not supported by their data and must be removed.

We agree with the reviewer that the portion of immigrants from geographical areas with more virulent genotypes could influence the clinical manifestations of the disease, but we have no information on pregnant women who were infected outside Spain, nor do we have information on the genotypes causing congenital toxoplasmosis in our cohort. As mentioned in the manuscript, it is assumed that the predominant genotype in Spain is genotype 2.

The sentence in lines 286-287 is consistent with the results derived from our study and we believe it is important to keep it, given that the majority of cases were detected by prenatal screening.

−       What could an obstetrician drive to undertake amniocentesis at week 38 when the game is over for influencing pregnancy. Please report the standards on which amniocentesis is usually done (line 194).

In Spain, the current protocols of the various scientific societies involved in CT recommend performing amniocentesis when maternal infection is suspected in any trimester of pregnancy.

−       The term “ambispective” is not generally used and not understandable in the context of this heterogenous cohort.

The term “ambispective cohort” has been used more and more frequently in scientific articles over the last 5 years, with more than 25 citations/year in the “title and/or the abstract” in Pubmed. We think it reduces the number of words while keeping the meaning of the sentence.

Reviewer #2: Table 3 should be redone.
I have doubts about the multivariate analysis and the definitions of infected newborn used

According to the reviewer 1, there is uncertainty about the possible relationship between the duration of prenatal treatment and its possible effects on the fetus. Therefore, we have redone the statistical analysis and have identified three categories (lines 140-144):

- Prenatal treatment: defined as receiving one or both treatments during pregnancy for at least 28 days.

- Uncertain prenatal treatment: treatment lasting between 7 and 27 days.

- No treatment: treatment less than 7 days

As no treatment is not the same as only a few days of treatment, with unknown impact on the course of the fetal infection, we decided to exclude the uncertain prenatal treatment group from the statistical analysis. It is important to note that early discontinuation of prenatal treatment was explained by the end of pregnancy in all 8 cases of this group (lines 217-218). Interestingly, the beneficial effects of prenatal treatment remained unchanged when prenatal treatment of more than 7 days was included in the analysis in the group of treatment, as we mention in the Discussion (lines 331-334).

Reviewer #3: Results are clearly presented

--------------------

<b>Conclusions</b></br></br>
-Are the conclusions supported by the data presented?</br>
-Are the limitations of analysis clearly described?</br>
-Do the authors discuss how these data can be helpful to advance our understanding of the topic under study?</br>
-Is public health relevance addressed?</br></br>

Reviewer #1: −  It is not a failure, but an active decision not to treat (line 200).

Many thanks for the suggestion. We have changed the expression.

−       Repetitions from results in text and tables need to be removed, Given the low number of observations, 1-2 tables might do, table 3 should be removed given the lack of strength and standardisation of treatment, as outlined above.

We consider not to remove the bivariate analysis of clinical outcomes according to whether the mother received treatment or not (table 3), in order to show the importance of the positive trend in favor of prenatal treatment that was observed in all variables, although it was not statistically significant (except in case of “new IRC all”).

−       The impact of 1 year of postnatal treatment onto outcomes deserves to be discussed, given the relatively high number of cases developing secondary complications. Please add this point in the discussion (lines 310 ff).

We agree with the reviewer about the importance of the effects of postnatal treatment on clinical outcomes (infection-related complications) in infected children. Unfortunately, the characteristics of our study did not allow us to assess the appearance of new infection-related complications (IRC) during follow-up by controlling for the effect of postnatal treatment. We discussed this in the limitations of the article (lines 405-406).

In reference to IRC, the appearance of new episodes of retinochoroiditis was the most common complication during follow-up (5/56, 9%), a slightly lower incidence than that reported in European cohorts of children treated postnatally (17% of children after 3 years of follow-up, or 24% after 6 years of follow-up). Most likely, the mean follow-up of only two years underestimates the true extent of long-term CT morbidity, and in particular of recurrences of retinochoroiditis (lines 411-413).

Reviewer #2: The conclusions are sufficiently supported by the data presented
-Multivariate analysis should be checked

We have redone and checked the analysis and the results remain unchanged.

The authors sufficiently discuss how these data can be useful to improve knowledge on Toxoplasmosis and to improve the prevention strategy

Reviewer #3: The conclusions support the data presented.
Some suggestions/comments are introduced in the letter to the authors.

--------------------

<b>Editorial and Data Presentation Modifications?</b></br><br/>
Use  this section for editorial suggestions as well as relatively minor modifications of existing data that would enhance clarity. If the only modifications needed are minor and/or editorial, you may wish to recommend “Minor Revision” or “Accept”.

Reviewer #1: (No Response)

Reviewer #2: (No Response)

Reviewer #3: “Minor Revision”

--------------------

<b>Summary and General Comments</b></br></br>
Use  this section to provide overall comments, discuss strengths/weaknesses of the study, novelty, significance, general execution and scholarship. You may also include additional comments for the author, including concerns about dual publication, research ethics, or publication ethics. If requesting major revision, please articulate the new experiments that are needed.

Reviewer #1: In the absence of solid prospective evidence for screening and intrauterine infection prophylaxis, and, if too late, therapy of children with CT, a systematic collection of retrospective data, as done in the “REIV-TOXO Project: Results from the Spanish cohort of congenital toxoplasmosis (2015-2022)” is urgently needed. In so far, I congratulate the authors to their activities, which are highly appreciated. Nevertheless, the strength of each single of these studies is not sufficiently strong to convince political opinion makers regarding its cost-effectiveness, while efficacy is principally accepted. As such, the REIV-TOXO Project adds to existing data, while a significant inherent limitation of this project is its observational character, since neither diagnostic not therapeutic standards have been applied explaining the heterogenicity of their data as visible by the broad standard deviations. This should have strong impact on the interpretation of their data and induce a critical discussion, which I have not seen.

I fully agree that a systematic screening, and be it on a minimal scale of once per trimester, is justified, if this triggers a consequent maternal treatment until delivery which was not the case in this series. This limits the strength of data and possible conclusions based thereon. From a public health perspective, cost-effectiveness is to be shown. What are the costs of preventive measures and prophylactic therapy compared to the costs induced by infected individuals. Public health physicians have not necessarily an idea what CT could mean for the affected individual and its family. And their primary perspective is unfortunately a reduction of health care costs.

Thank you for your comments. We acknowledge the limitations of our observational and ambispective study, and we mention this in the Discussion. However, it does not mean that we should question or downplay the importance of the results obtained. The national CT cohort of REIV-TOXO provides updated information on the status of CT in Spain and the beneficial effects of prenatal treatment. The broad standard deviations are also explained by the limited sample size.

Our study has not addressed toxoplasmosis from the point of view of the cost and effectiveness of diagnosis, screening and treatment of the disease. This is not the aim of our work. However, we fully agree with your opinion and we should present our results in order to generate cost-effectiveness studies to help public health decision-making.

Reviewer #2: PLOS Neglected Tropical Diseases
REIV-TOXO Project: Results from the Spanish cohort of congenital toxoplasmosis (2015-2022)
It is known that toxoplasmosis contracted during pregnancy can cause congenital infection of the newborn and serious outcomes. The burden of this disease in the EU/EEA cannot be assessed due to large differences in national surveillance systems, screening programs and follow-up procedures of pregnant women. In addition to implementing national surveillance strategies, prevention of congenital toxoplasmosis should be implemented. Pregnant women should always receive information on the risks of exposure to T gondii and on the preventive measures to be taken. Serological screening during pregnancy, given that there is an effective therapy, should be implemented. The manuscripts that provide data are very relevant.

Author summary, page 5 lines from 67 to 70
Authors: We also focused on the effect of prenatal treatment on clinical outcomes at birth and during follow-up, since its effectiveness is still controversial and may raise several concerns. One of these issues is the actual benefit of serological screening during pregnancy as part of a public health strategy to mitigate the disease.

Reviewer: I believe that no one can contradict the usefulness of carrying out prenatal screening for toxoplasmosis during the pregnancy, given that there is a therapy which, regardless of the data collected in Spain, is proven to prevent the onset of feto-neonatal damage or in any case limit its severity. I would say to rephrase the sentence.

We have rephrased the sentence accordingly (line 71).

We agree with the reviewer that the available evidence, derived from more than 30 observational studies involving more than 9000 pregnant women and children, seems to indicate that prenatal treatment can reduce mother-to-child transmission and sequelae in infected children. But in the early 2000s, routine screening and treatment for toxoplasmosis during pregnancy became controversial because of articles that were published from 1999 to 2007, that unfortunately were biased and misleading and misunderstood the biologic impact of antiparasitic treatments in pregnancy. Therefore, we have added this to the manuscript.

Page 5 line 71
Authors: Although the usefulness of prenatal screening is still under debate, …..
Reviewer: there is a therapy which is proven to prevent the onset of damage in fetuses and in neonates or in any case limit its severity. Please rephrase the sentence.

We have changed it to “implementation” (line 73).

Authors: prenatal screening is still under debate, it remains the only tool available to diagnose all children …….
Reviewer: Diagnosis of infection in pregnancy is not a tool for diagnosing infection in children.... please rephrase.

We have changed this to “ the only tool available that allows any potentially infected children to be identified...” (line 74)

Page 6 line 96
Authors: At present, prenatal serological screening is the only diagnostic tool available to identify all …….
Reviewer: as mentioned above the sentence is improper. Prenatal screening identifies infected mothers. Please rewrite the sentence.

We changed it to “the only tool available that allows any potentially infected children to be identified...”

Page 6 line 98
Reviewer: Please add some other informations “Toxoplasmosis in pregnancy is generally asymptomatic and the diagnosis is based only on serological tests.  Some Countries such as France Austria Italy promote regular serological screening of pregnant women. Other countries have no prenatal surveillance program at all. The reasons for these differences are multiple and debatable. These include the effectiveness of prenatal treatment and the cost-effectiveness of such a program. Nevertheless, a significant body of data has demonstrated that rapid onset of treatment after maternal infection reduces the risk and severity of fetal infection.

We agree with the recommendations of reviewers 2 and 3. We have added more information on the various strategies in different European countries and the main argument for discontinuing screening (lines 102-109).

Page 8 line 125
Authors: The inclusion criteria for a confirmed CT case in the REIV-TOXO registry …..
Reviewer: The precise wording would be:
"Newborns who showed: positivity of ....." were defined as newborns affected by congenital Toxoplasmosis, symptomatic or non-symptomatic.
Newborns defined as having Congenital Toxolasmosis were reported through the registry....

It would be best to identify a separate paragraph within the methods section, which contains definitions and then state who was reported through the registry.

We have modified this paragraph in the Methods section for better understanding (lines 134-139).

Page 9 lines 156-157
Authors: The same model was also used to compare the two regimens received: SPI and PSA, with SPI and/or PSA treatment defined as treatment received for at least seven days.
Reviewer: It would also be interesting to know the effect on the infectious status of the newborn at the gestational age at which antiprotozoal therapy began, because Toxoplasma crosses the placenta more in the last trimester of gestation.

We agree with your comment. That is why we performed the multivariate logistic regression model, adjusted for the type of treatment in the pregnant mother and for the time of maternal infection, to control for potential effects of the latter (lines 171-173).

Page 11 lines 193-195

Authors: Amniocentesis was performed in a third (18/49; 36.7%) of the pregnant women, with the procedure done at a median gestational age of 29 weeks (IQR, 20–38) with PCR for T. gondii in AF being positive in 12/18 cases (66.7%) …

Reviewer: Why amniocentesis so late? Why such a low number of cases during the pandemic? Please, discuss these particularities.

We agree with the reviewer about the low number of cases of amniocentesis. Unfortunately, the reasons for not performing amniocentesis are not included in the REIV-TOXO registry. Accordingly, we mention in the Discussion that performing amniocentesis is an area for future improvement (lines 374-376).

As we discussed previously, current protocols recommend performing amniocentesis when maternal infection is suspected in any trimester of pregnancy. Most notably, it is not contraindicated in late pregnancy, and the safety of the procedure should be carefully evaluated by the prenatal diagnosis specialist. Late amniocentesis corresponds to the diagnosis of gestational toxoplasmosis in the third trimester.

Page 16 lines from 266 to 268
Authors: A statistically significant association (p = 0.01) was also observed between prenatal treatment and the absence of neurological involvement at birth, with a 4.65-fold risk of developing neurological symptoms in newborns whose mothers did not receive antiparasitic treatment (OR, 4.65; 95% CI, 1.28–16.78).
Reviewer: It would be better to express the association between the absence of maternal therapy and feto-neonatal neurological damage. How can we explain the lack of significance of the multivariate association in table 3?
Is it necessary to recheck the case definition? In Europe all the reporting Member States used the EU case definition from 2008, 2012, or 2018 (the case definition remained the same), except two which used other (not specified) case definitions.

We attributed the lack of significance of the multivariate analysis to the low sample size with insufficient power to detect differences. A positive trend in favor of prenatal treatment was observed in all variables, although it was not statistically significant (except in case of “new IRC all”). This is the reason for maintaining all data in table 3.

We have adopted the case definition established in our local protocol, in line with the established criteria in the literature:

**Confirmed congenital Toxoplasma infection** – Congenital infection is confirmed by any of the following:

1-Positive IgG with positive IgM and/or IgA – A positive *Toxoplasma* IgM and/or IgA

2-Positive polymerase chain reaction (PCR) in blood, CSF, urine, amniotic fluid or placenta.

3-Increase in anti-*Toxoplasma* IgG titer during the first year of life

4-Positive IgG beyond 12 months of age

In summary the manuscript is very important because it provides neonatal data on a topic on which there is fragmentary epidemiological knowledge. The data in the table should be rechecked for the association between the dependent variables (symptoms, neonatal neurological damage, ocular damage) and the risk factor "absence of treatment during pregnancy".
To be re-evaluated after corrections.

We appreciate all your comments. We have redone the statistical analysis in line with comments of the reviewer 1 and have identified three categories (lines 140-144):

-Prenatal treatment: defined as receiving one or both treatments during pregnancy for at least 28 days.

-Uncertain prenatal treatment: treatment lasting between 7 and 27 days.

-No treatment: treatment less than 7 days

Because no treatment is not the same as a few days of treatment, with unknown impact on the course of the fetal infection, we decided to exclude the uncertain prenatal treatment group from the statistical analysis.

As we explained earlier, the association between “neonatal neurological damage” and “ocular damage”, and the risk factor "no treatment during pregnancy” was rechecked with the new analysis and demonstrated that the beneficial effects of prenatal treatment remained unchanged. Also, a positive trend in favor of prenatal treatment was observed in all variables in the multivariate analysis.

Reviewer #3: The paper titled "Results from the Spanish cohort of congenital toxoplasmosis (2015-2022)" by Guarch-Ibanez et al. highlights the importance of maternal screening for T. gondii in Spain and treatment for the management of congenital toxoplasmosis. The conclusions drawn from this paper support the implementation of a mandatory prenatal screening program followed by treatment for women who test positive to reduce the impact of the disease on newborns and during follow-up. Here, we applaud the authors for citing on many occasions France, a country with historically successful management of congenital toxoplasmosis, and a similar incidence rate of CT in Spain, which suggests that prenatal screening is cost-effective compared to neonatal screening. This approach also empowers pregnant women to educate themselves on how to reduce the risk of infection and decrease the incidence of toxoplasmosis in the country. However, the data from the Spanish cohort lacks the monthly serological screening of pregnant women, which creates uncertainty about the timing of maternal infection before diagnosis and the potential delay in treatment following seroconversion. Despite this, the paper is well-written and interesting to read. I recommend that the authors emphasize these key points to enhance the presentation of this manuscript.
1. The current title of the manuscript does not accurately represent its content and should be changed.

We suggest this title:

REIV-TOXO Project: Results from a Spanish cohort of congenital toxoplasmosis (2015-2022). The beneficial effect of prenatal treatment on clinical outcomes of infected newborns.

2. The introduction and conclusion of the manuscript need further development. Please provide more details on why certain European countries (such as the UK, Denmark, and Sweden) and specific regions in Spain have rejected prenatal screening. Some argue that this decision is due to the clinical harms associated with false-positive diagnoses, the low and uncertain benefits of treatment, the harms associated with treatment, and the high costs of prenatal screening.

We have added the main arguments used to justify nonimplementation or withdrawal of prenatal screening to the Introduction (lines 104-107). The specific arguments for withdrawal of prenatal screening in Spain are discussed in a previous article from our group [24]. We did not want to discuss the arguments in depth, in order to avoid making the discussion too long.

3. Are there any studies from the Spanish cohort on the genetics of the parasite that could explain the severity observed in certain newborns, such as the case of a newborn with hydrocephalus?

The parasite genotype of the cases included in REIV-TOXO is unknown. This is due to the characteristics of the cohort (retrospective and prospective) and the difficulties of genotyping the parasite, which is only possible in a very few specialised reference laboratories. It is assumed that the genotype of the parasite is probably genotype II, the most prevalent one in Europe.

4. Technical information is necessary to understand how the diagnosis and screenings of pregnant women were reported in this cohort. Was the avidity test used to confirm those who are seropositive and not required for the screening? Which confirmation test for IgG/IgM was used? Do all these hospitals use the same confirmatory test? Is there any consensus on the choice of this test?

REIV-TOXO collects laboratory results from more than 30 hospitals in Spain. Most clinical laboratories use chemiluminescent immunoassays (CLIA) and, less frequently, enzyme immunoassays (ELISA, ELFA). All of them are generally specific and sensitive tests.

In Spain, prenatal screening for toxoplasmosis is performed by systematic determination of specific IgG against T. gondii:

- In the case of a negative IgG result, a follow-up is performed by IgG retesting every trimester.

- In the case of a positive IgG result, specific IgM against T. gondii is performed. If IgM is negative, the pregnant woman is considered immune and serological follow-up is not continued. In the case of a positive IgM result, the IgG avidity test is performed. Referral to a reference laboratory is recommended for verification by another serological technique not previously used, and in inconclusive cases, Western-blot for IgG and IgM confirmation is performed.

5. Please provide information on whether pregnant women who tested positive for toxoplasmosis had any adverse pregnancy outcomes, such as miscarriages or preterm births. Did you observe any differences in outcomes between toxoplasmosis-positive and negative women or between those who received treatment and those who did not?

One of the limitations of our cohort is that it does not record abortions or stillbirths; this is explained in the part of limitations of the manuscript. The manuscript also mentions that most patients with CT were born at term (85.7%), and only 14.3% were preterm births.

We don’t have data from toxoplasmosis-negative mothers, only positive or unknown (in case of no follow-up). Our results confirm the beneficial effect of prenatal treatment on children whose mothers received prenatal treatment compared with those who did not.

6. The authors should provide evidence to support their claim about the impact of SARS-CoV-2 on the decrease in reported cases of T. gondii infection in mothers in Spain since 2018 and whether similar trends are seen in other congenital diseases. Please include any relevant references from the literature.

We added this reference (29): Villaverde S, Esquivel E, Baquero-Artigao F, Noguera-Julian A, Frick MA, Rojo P, Blázquez-Gamero D; on behalf of the Spanish Registry of Children with Congenital CMV. Impact of the COVID-19 Pandemic on the Diagnosis of Congenital Cytomegalovirus Infection in Spain. Pediatr Infect Dis J. 2022;41(7):590-592.

We have readded citation number 59, which was not contained in the previous version of the manuscript.

7. It would be helpful to enhance the discussion by emphasizing the importance of new diagnostic tests (e.g. rapid tests) that can be incorporated into prenatal screening to alleviate the challenges of monthly screening in a prospective study of this cohort.

We agree with you. We now mention the importance of including POC tests in prenatal screening (ref 50-51). You can find this on lines 358-360.
--------------------

PLOS authors have the option to publish the peer review history of their article (<a href="<https://journals.plos.org/plosntds/s/editorial-and-peer-review-process#loc-peer-review-history>" target="_blank">what does this mean?</a>). If published, this will include your full peer review and any attached files.<br><br>

If you choose “no”, your identity will remain anonymous but your review may still be made public.<br><br>

<b>Do you want your identity to be public for this peer review?</b> For information about this choice, including consent withdrawal, please see our <a href="<https://www.plos.org/privacy-policy>" target="_blank">Privacy Policy</a>.

Reviewer #1: Yes: Justus G. Garweg

Reviewer #2: No

Reviewer #3: Yes: KAMAL EL BISSATI


Figure Files:
While revising your submission, please upload your figure files to the Preflight Analysis and Conversion Engine (PACE) digital diagnostic tool, [https://pacev2.apexcovantage.com](https://pacev2.apexcovantage.com/). PACE helps ensure that figures meet PLOS requirements. To use PACE, you must first register as a user. Then, login and navigate to the UPLOAD tab, where you will find detailed instructions on how to use the tool. If you encounter any issues or have any questions when using PACE, please email us at figures@plos.org.
 
Data Requirements:
Please note that, as a condition of publication, PLOS' data policy requires that you make available all data used to draw the conclusions outlined in your manuscript. Data must be deposited in an appropriate repository, included within the body of the manuscript, or uploaded as supporting information. This includes all numerical values that were used to generate graphs, histograms etc.. For an example see here: <http://www.plosbiology.org/article/info%3Adoi%2F10.1371%2Fjournal.pbio.1001908#s5>.
 
Reproducibility:
To enhance the reproducibility of your results, we recommend that you deposit your laboratory protocols in protocols.io, where a protocol can be assigned its own identifier (DOI) such that it can be cited independently in the future. Additionally, PLOS ONE offers an option to publish peer-reviewed clinical study protocols. Read more information on sharing protocols at <https://plos.org/protocols?utm_medium=editorial-email&utm_source=authorletters&utm_campaign=protocols>

__________________________________________________
In compliance with data protection regulations, you may request that we remove your personal registration details at any time.  (Use the following URL: <https://www.editorialmanager.com/pntd/login.asp?a=r)>. Please contact the publication office if you have any questions.
