# Supplementary material for: REIV-TOXO Project: Results from a Spanish cohort of congenital toxoplasmosis (2015–2022). The beneficial effects of prenatal treatment on clinical outcomes of infected newborns
Source: PLoS Negl Trop Dis. 2024 Oct 22;18(10):e0012619. doi: 10.1371/journal.pntd.0012619 (PMC11530059; doi:10.1371/journal.pntd.0012619)
Supplement: Attachment — Submitted filename: Guarch-Ibanez et al. paper_PNTD 2024 _response reviewers II.docx [file pntd.0012619.s005.docx]

Reviewer's Responses to Questions

In this document we gather our responses to all reviewer´s comments, they are made in blue after every comment or question.

**Methods**

Reviewer #1: Methodological weakness needs acknowledgement in the manuscript.
The exclusion of 16 children with confirmed CT from the registry limits representativity as the authors state. The explanation given to the reviewer should be added to the manuscript: “REIV-TOXO is a voluntary registry, where the investigators at each site are responsible for entering the data, limit the authors' access to all cases, and thus there are 16 cases that were never entered despite our efforts to contact the collaborators on repeated occasions. Additionally, without parental consent, we are unable to provide this information. Despite this, our registry represents the highest number of cases registered in Spain, much higher than those reported by the public health authorities.”

Thank you for your comments. We would like to note that the proposed explanation is already included in the study's limitations (lines 397-401). Additionally, we have revised the Conclusions section to incorporate the final sentence of your suggestion: “Despite all this, our registry represents the highest number of cases registered in Spain, much higher than those reported by the public health authorities” (lines 405-406).

**Results**

Reviewer #1: The requested information regarding regional data about the parasite strain types (which type is prevalent in Spain, 1, type 2, recombinant strains) in human disease. What is the prevalence of seropositivity in pregnant women, and what was the number of recorded life births with CT per year during the same period? These points have remained unanswered and deserve to be added to the manuscript. Genotyping data of given patients were not requested.

As we mentioned previously, the parasite genotype for the cases included in REIV-TOXO is unknown. This is due to the characteristics of the cohort (both retrospective and prospective) and the difficulties of genotyping the parasite, which can only be performed in a limited number of specialized reference laboratories.

To date, there are very few studies of genomic characterization of Toxoplasma isolates in Spain but, at least in the general population, the type II genotype is assumed to be the most frequent (J Clin Microbiol. 2001 39:1566-70), as is the case in the rest of Europe, being this data confirmed by studies carried out on animals (J Parasitol. 2008; 94:1044-6 and Front Cell Infect Microbiol. 2022;12:842595). This point is already mentioned in the Introduction section (lines 94-95) and we have added these 3 references additional to the manuscript.

Regarding pregnant women, a study recently published by one of the authors of this manuscript reported that, although a decrease in prevalence has been identified, the pooled seroprevalence of toxoplasmosis in pregnant women in Spain was 24.4% (95% CI 21.2-28.0%) (J Epidemiol Glob Health. 2024 Jun 12. doi: 10.1007/s44197-024-00258-w. Epub ahead of print. PMID: 38864976) We have added this information and reference to the manuscript as suggested by the reviewer.

Finally, the number of recorded life births with CT per year during the period of the study, is already mentioned in the Introduction section (lines 117-119).
